# Seagrass‐associated fungal communities show distance decay of similarity that has implications for seagrass management and restoration

**DOI:** 10.1002/ece3.5631

**Published:** 2019-09-15

**Authors:** Benjamin J. Wainwright, Geoffrey L. Zahn, Joshua Zushi, Nicole Li Ying Lee, Jillian Lean Sim Ooi, Jen Nie Lee, Danwei Huang

**Affiliations:** ^1^ Department of Biological Sciences National University of Singapore Singapore City Singapore; ^2^ Biology Department Utah Valley University Orem UT USA; ^3^ Department of Geography Faculty of Arts and Social Sciences University of Malaya Kuala Lumpur Malaysia; ^4^ Faculty of Science and Marine Environment University Malaysia Terengganu Terengganu Malaysia; ^5^ Tropical Marine Science Institute National University of Singapore Singapore City Singapore

**Keywords:** biodiversity, biogeography, conservation, dispersal, *Enhalus acoroides*, marine fungi, Southeast Asia

## Abstract

Marine fungal biodiversity remains vastly understudied, and even less is known of their biogeography and the processes responsible for driving these distributions in marine environments. We investigated the fungal communities associated with the seagrass *Enhalus acoroides* collected from Singapore and Peninsular Malaysia to test the hypothesis that fungal communities are homogeneous throughout the study area. Seagrass samples were separated into different structures (leaves, roots, and rhizomes), and a sediment sample was collected next to each plant. Amplicon sequencing of the fungal internal transcribed spacer 1 and subsequent analysis revealed significant differences in fungal communities collected from different locations and different structures. We show a significant pattern of distance decay, with samples collected close to each other having more similar fungal communities in comparison with those that are more distant, indicating dispersal limitations and/or differences in habitat type are contributing to the observed biogeographic patterns. These results add to our understanding of the seagrass ecosystem in an understudied region of the world that is also the global epicenter of seagrass diversity. This work has implications for seagrass management and conservation initiatives, and we recommend that fungal community composition be a consideration for any seagrass transplant or restoration programme.

## Graphical abstract

Examining marine fungi associated with the seagrass, *Enhalus acoroides*.

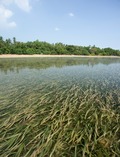

## 1 INTRODUCTION

Globally, the biogeography of fungi is not well understood (Krah, Bates, & Miller, 2019; Tedersoo et al., 2014; Tisthammer, Cobian, & Amend, 2016). In marine environments, where even studies of fungal biodiversity are in their infancy, our understanding of fungal biogeography is rudimentary (Amend et al., 2019; Wainwright et al., 2017; Wang, Wang, Liu, & Li, 2012). However, steps to address this are afoot (http://www.marinefungi.org; Jones et al., 2019). The current lack of research focus on marine systems is unfortunate given our residence on a planet that has over 70% of its surface covered in saline water, more so since these oceans contain one of the largest organic carbon pools on earth and have a central role in climate regulation and supporting life on our planet (Gattuso et al., 2018; Moran et al., 2016). Nevertheless, we do know that fungi play a vital and active role in oceanic carbon cycling (Wang et al., 2012); consequently, studies of marine fungal diversity and distributions are especially pertinent to fully understand how ocean systems will respond to climate change. This limited understanding is concerning since fungal distributions, diversity, and functioning can be altered dramatically by anthropogenic stressors, especially those driven predominantly by climate change (Epp Schmidt et al., 2017). Here, we examine the fungal communities associated with the seagrass *Enhalus acoroides* throughout Singapore and Peninsular Malaysia to test the hypothesis that fungal communities are homogeneous throughout all study sites on account of their assumed high dispersal potential (Raghukumar, 2017).

Seagrasses are the descendants of terrestrial plants that made the transition from land to marine habitats beginning ~100 million years ago (Olsen et al., 2016). Presently, they are found on all continents other than Antarctica. Seagrasses are capable of forming large meadows that provide numerous critical ecological services. For example, they act as vital nursery habitat for many coral reef species (e.g., fish and invertebrates; Harborne et al., 2006; Unsworth, Nordlund, & Cullen‐Unsworth, 2019), reduce wave energy, help trap sediment, and prevent its resuspension thereby increasing water clarity that is essential to coral survival (Christianen et al., 2013; Waycott et al., 2009). Seagrasses play a vital role in carbon sequestration where carbon dioxide used in photosynthesis helps mitigate climate change and ocean acidification; it is estimated that seagrasses are responsible for nearly 15% of total global carbon storage (Kennedy & Bjork, 2009). Additionally, work by Lamb et al. (2017) details the active role that seagrasses play in reducing human contact with bacterial pathogens and the general role they play in water filtration, ultimately improving water quality.

Increasingly, seagrass meadows are suffering the detrimental effects of multiple stressors (e.g., overexploitation, physical modification, nutrient and sediment pollution, invasive species and global climate change), and seagrass losses are accelerating (Waycott et al., 2009). These declines are particularly acute in Southeast Asia, the global epicenter of seagrass biodiversity (Fortes et al., 2018). The seagrasses from this region are also some of the least‐studied in the world and considerable gaps in basic knowledge such as species distributions are common (Fortes et al., 2018; Waycott et al., 2009).

To curb further seagrass losses, numerous management plans have been implemented, these include efforts to reduce anthropogenic impacts from controlling and minimizing land‐based, point source pollution and eutrophication, to establishing best practices for preventing mechanical damage from boat propellers and anchors (Waycott et al., 2009). To restore habitats, seagrass transplantation and the planting of seeds are becoming increasingly common, yet these approaches are difficult and <40% of transplantation schemes are actually successful, a result of the dynamic and stressful environment in which seagrasses grow (van Katwijk et al., 2015).

Under terrestrial habitat restoration schemes, which are relatively common (Miller & Hobbs, 2007), studies have shown that fungal communities found in the restoration or transplant location are critical predictors of success (Berruti, Lumini, Balestrini, & Bianciotto, 2015). In particular, host–pathogen resistance can be increased by matching symbiotic fungal communities as closely as possible to communities found in areas where the host is healthy (Zahn & Amend, 2017). Similarly, to maximize the success of marine transplantation and restoration initiatives, we suggest that fungal communities should be a key consideration for source site selection, with transplants ideally coming from locations that have similar fungal community compositions. Seagrass‐associated pathogens have been found in the Atlantic, Mediterranean, and Gulf of Mexico where they have negatively impacted ecosystem functioning and conservation efforts (Govers et al., 2016; van Bogaert et al., 2019). Therefore, detailed characterization of the microbial communities and associated microbiomes of seagrasses may help prevent the accidental introduction of harmful pathogens to areas that are pathogen‐free.

By examining the fungal communities associated with the widespread and easily identifiable seagrass *E. acoroides* in Southeast Asia, we aim to advance our knowledge of marine fungal biodiversity and biogeography. At the same time, this work allows us to increase our understanding of seagrasses and their associated microbial communities in a marine biodiversity hotspot. Importantly, these results can be leveraged to improve the success of future seagrass restoration and transplantation efforts.

## 2 METHODS

**Figure 1:**
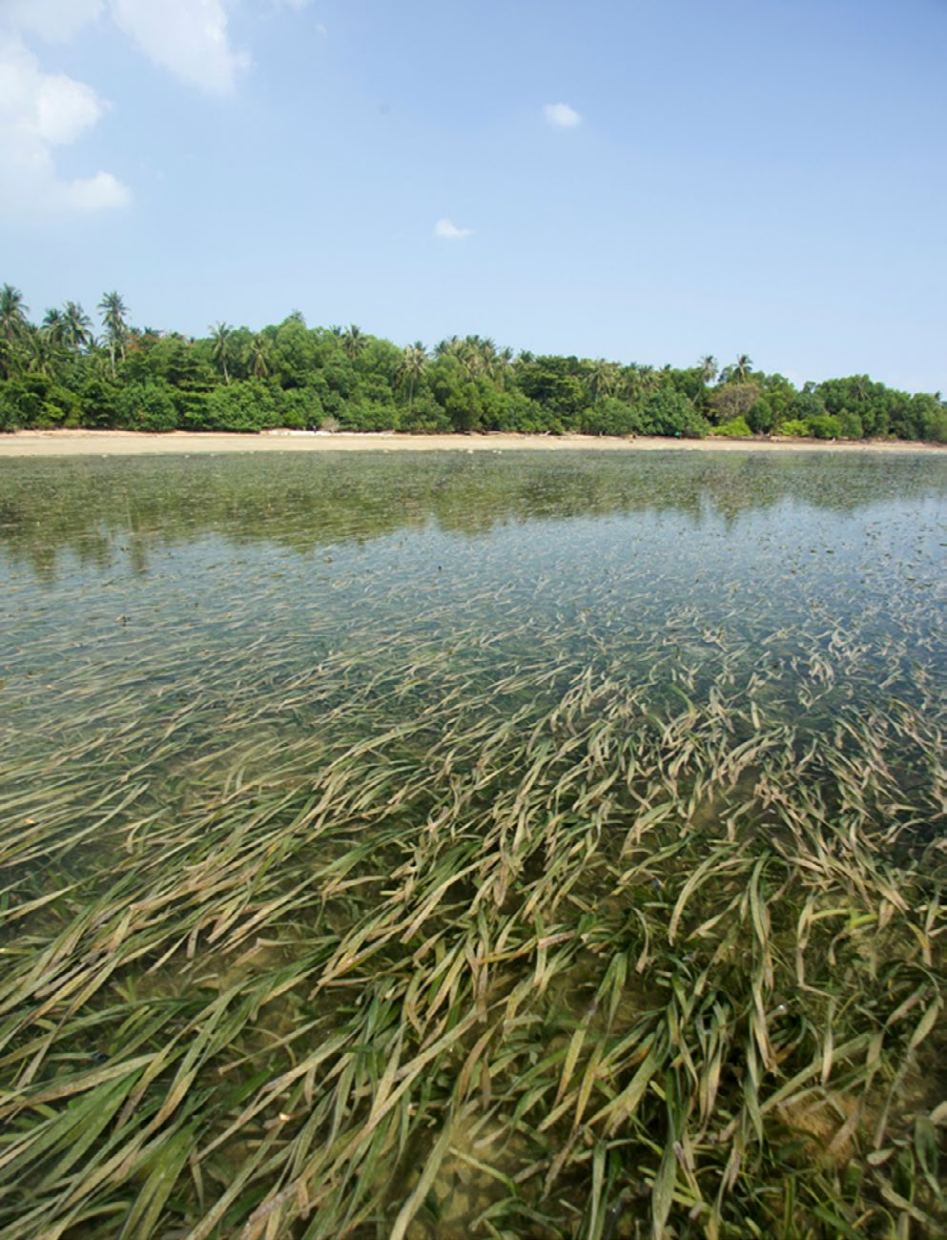
Seagrass meadow primarily composed of the seagrass, *Enhalus acoroides*. Picture taken at Pulau Semakau, Singapore. (photo credit: © Ria Tan, http://wildsingapore.com)

**Figure 2:**
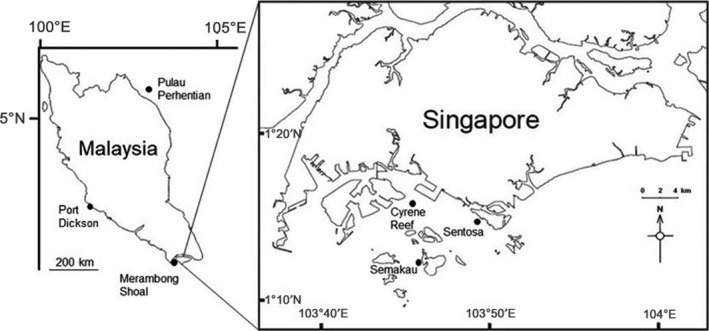
Map showing the location of sampling sites throughout Singapore and Malaysia

Ten complete, unconnected *E. acoroides* plants (Figure 1), free of any visible epiphytes were collected from a total of six locations, three in Singapore and three in Malaysia. (Figure 2). Each plant was separated by at least 10 m, and all collections were made at low tide. Using a sterile razor blade, individual plants were separated into leaves, roots, and rhizomes. Additionally, one sediment sample was taken in close proximity (<1 m) to each collected plant using a syringe placed approximately 4 cm below the surface. All seagrass tissues were surface sterilized by immersion in 1% NaClO for 2 min, 70% EtOH for 2 min and rinsed twice in sterile autoclaved water for 5 min. Tissues were disrupted in an Omni Bead Ruptor 24 (Omni International) at 8 m/s for 2 min.

DNA was extracted with a Qiagen DNeasy Powersoil kit following the manufacturer's instructions. Because the mass of host DNA will be several orders of magnitude greater than that of fungal template, DNA concentration was not quantified. Fungal DNA amplification of the internal transcribed spacer 1 (ITS1) region was performed using the ITS1F (CTT GGT CAT TTA GAG GAA GTA A; Gardes & Bruns, 1993) and ITS2 (GCT GCG TTC TTC ATC GAT GC; White, Bruns, Lee, & Taylor, 1990) primers, which were modified to include Illumina adaptors, a linker and a unique barcode for each forward and reverse primer (see Smith & Peay, 2014 for details of custom sequencing primers). Each reaction was performed in a total volume of 25 µl, containing 9 µl of template, with final concentrations of 0.25 U of KAPA 3G Enzyme (Kapa Biosystems, Inc.), 0.3 µM of each primer, 1.5 mg/ml of BSA and KAPA Plant PCR Buffer to 25 µl. PCR cycling protocol was 95°C for 3 min, followed by 35 cycles of 95°C for 20 s, 53°C for 15 s, 72°C for 20 s with a final extension at 72°C for 60 s. Negative PCR and extraction blanks were included to identify any possible contamination issues. PCR products were visualized on a 1% TBE buffer agarose gel, then normalized and cleaned using SequalPrep™ normalization plates (Invitrogen). Purified PCR products were submitted for sequencing on the Illumina MiSeq platform (600 cycles, V3 chemistry, 300‐bp paired‐end reads) with a 15% PhiX spike at the Genome Institute of Singapore (GIS).

The ITS1 region of the rDNA was extracted from all sequenced amplicons using ITSxpress (Rivers, Weber, Gardner, Liu, & Armstrong, 2018). Reads were then quality screened and reverse reads were discarded, a strategy that frequently improves results (Pauvert et al., 2019). Forward reads were processed in R using the *DADA2* package (Callahan et al., 2016) to filter any reads with uncalled bases or with a max EE value of 2 (see https://benjjneb.github.io/dada2/ for a detailed explanation of filtering parameters). Quality‐filtered reads were then used to estimate and correct sequencing errors, and remove de novo‐detected chimeras within the *DADA2* package. Contaminant sequences found in negative controls were removed using the prevalence method in the *decontam* R package (Davis, Proctor, Holmes, Relman, & Callahan, 2018). Cleaned and filtered Exact Sequence Variants (ESVs) were then assigned taxonomy with the RDP Classifier algorithm against a custom database consisting of the UNITE database (v. 1.12.2017) and a custom set of outgroups including *Enhalus* and anthozoan ITS1 sequences (available at: https://github.com/gzahn/Enhalus_Fungi/tree/master/Taxonomy). Any sequences matching nonfungal taxa were removed. The remaining ESVs that were taxonomically assigned as fungi were used in all downstream analyses within the *phyloseq* R package (McMurdie & Holmes, 2013).

Absolute ESV counts from each sample were transformed to relative abundance values to account for the compositional nature and sequence heterogeneity inherent in Illumina datasets (Gloor, Macklaim, Pawlowsky‐Glahn, & Egozcue, 2017). A Mantel test with 999 permutations was performed between community and geographic distance matrices with the mantel.rtest function in the *ade4* package (Bougeard & Dray, 2018). Additionally, we employed multiple regression on distance matrices with 9,999 permutations in the *ecodist* package to complement and confirm Mantel test results.

To determine which factors (location and/or plant part) structured fungal community, a permutational multivariate analysis of variance (PermANOVA) test was performed using the adonis function in the *vegan* package with 999 permutations (Okansen et al., 2016). Weighted classical multidimensional scaling was also performed in the *vegan* package. Heatmaps were generated with R and Venn diagrams were built using the *VennDiagram* R package (Chen, 2018). Network analyses were generated with the *igraph* package using presence–absence transformed data and a maximum distance of 0.9.

All analysis code and outputs for this project, including our taxonomic reference database can be found at https://github.com/gzahn/Enhalus_Fungi, and all raw sequences associated with this work have been deposited at the National Centre for Biotechnology Information under the BioProject Accession PRJNA517736.

## 3 RESULTS

Our analyses of fungal communities associated with the seagrass *E. acoroides* in Singapore and Peninsular Malaysia show that hosts sampled from different localities harbor significantly different fungal communities (*p* < .01; Table S1). Rarefaction curves showed that all samples reached asymptote (Figure S1), indicating adequate sequencing depth to capture fungal community diversity, although the read depth varied from sample to sample (see Table S2 for basic sequencing statistics).

**Figure 3:**
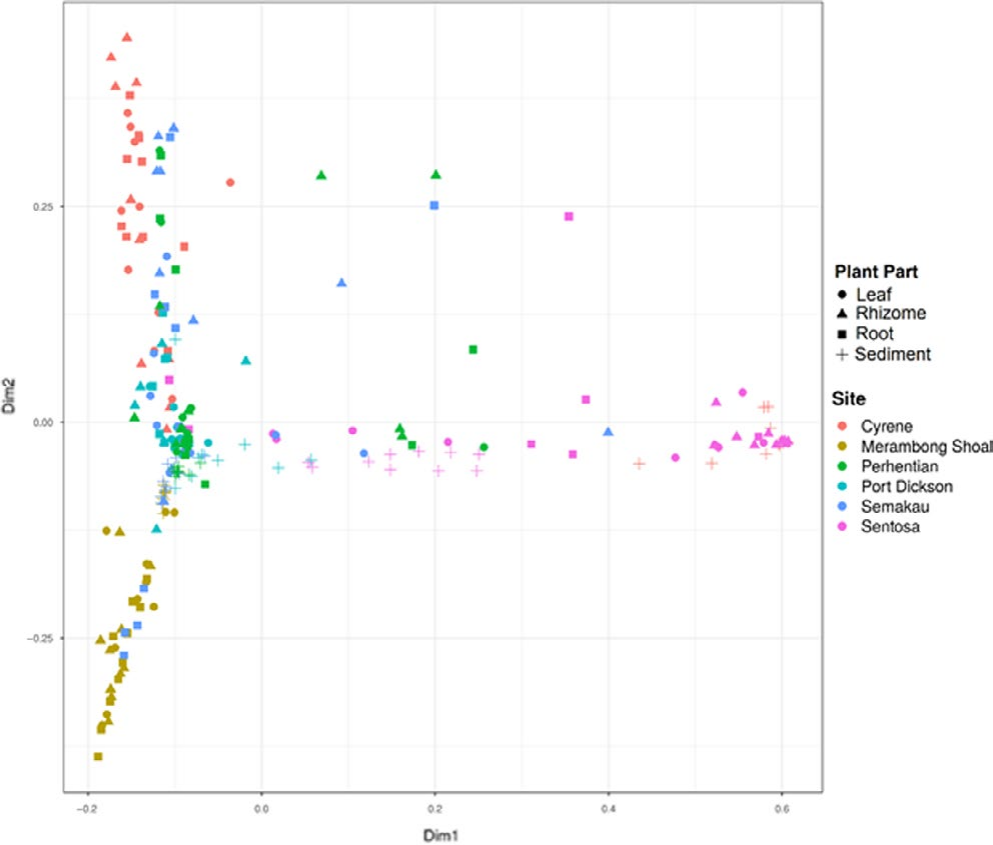
Weighted classical multidimensional scaling plot, colored by region, symbols represent plant part DNA was extracted from. Goodness‐of‐fit statistic = 0.106; based on Bray–Curtis distance of relative abundance values

**Figure 4:**
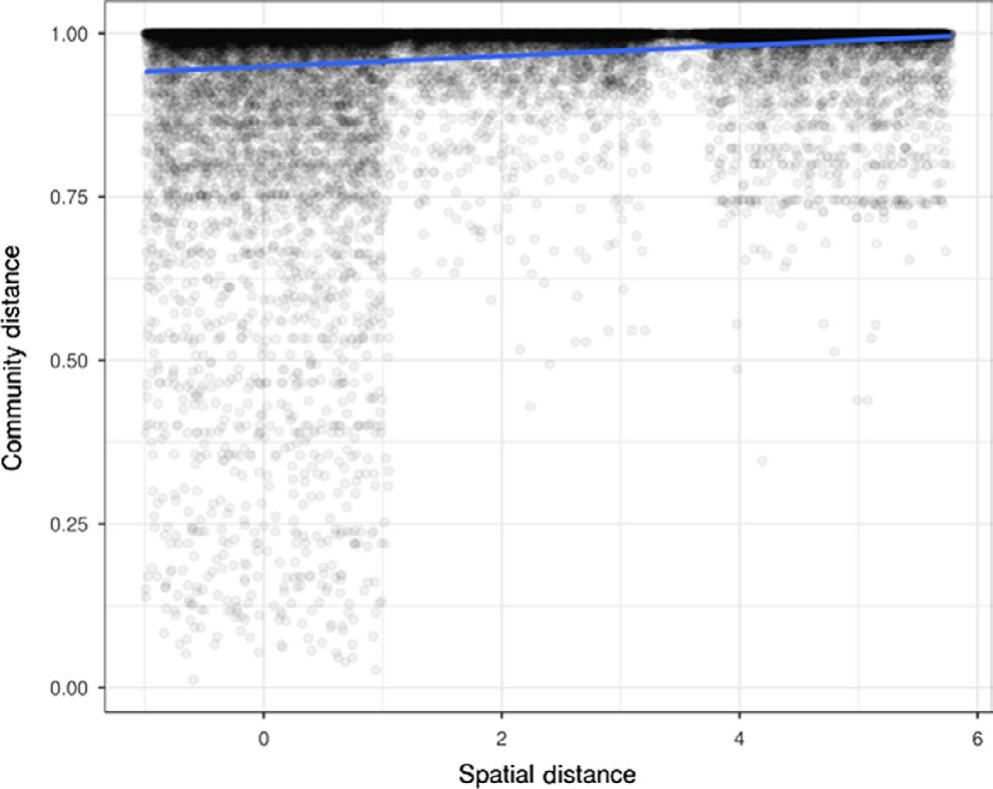
Mantel plot indicating a weak but significant positive distance–decay relationship (*r* = .16, simulated *p* < .01, based on 999 replicates)

Two samples, one root from Port Dickson, and one leaf from the Perhentian Islands failed QC and were removed from subsequent analyses (Table S3). Weighted classical multidimensional scaling ordinations show community structuring at the country level with samples from western Peninsular Malaysia and Singapore forming separate, relatively well‐defined clusters, while samples from eastern Peninsular Malaysia overlap with communities from both western Peninsular Malaysia and Singapore (Figure S2). At a smaller spatial scale, there appears to be clustering of fungal communities within each country by sampling location, especially those collected from Cyrene (Singapore) and Merambong Shoal (western Peninsular Malaysia; Figure 3). The same plots suggest further fungal community structuring by sampled source (leaf, rhizome, root, or sediment) with sediment samples tending to cluster by country and sampled location (Figure 3 & Figure S2). This structuring is confirmed by the results of PermANOVA, indicating that both sampling location and plant part (leaf, root, rhizome, and sediment) have significantly different fungal communities (*p* < .01; Table S1). Mantel test indicates a weak but significant positive relationship between geographic distance and community structure (*p* < .01; Figure 4 & Table S4); in other words, samples that are closer in proximity have more similar fungal communities. Further, multiple regression analyses on distance matrices showed the same pattern (MRM; 9,999 permutations; *p* < .01; Table S5). Additionally, redundancy analysis (RDA) shows that location is one of the primary variables responsible for differences in fungal community structure (Figure S3).

**Figure 5:**
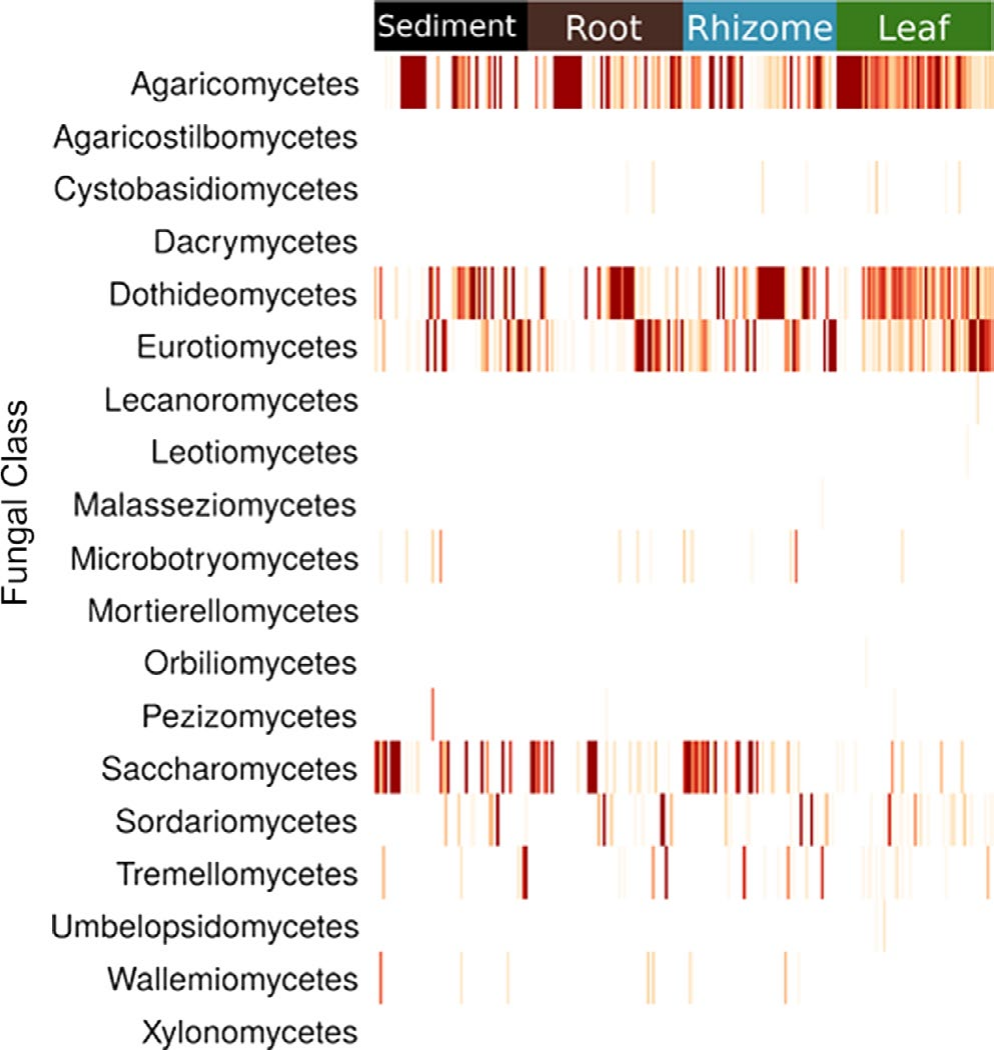
Heatmap of relative abundance of fungal class in each sample. Samples are grouped by the plant structure they were taken from. Deeper red indicates higher abundance

**Figure 6:**
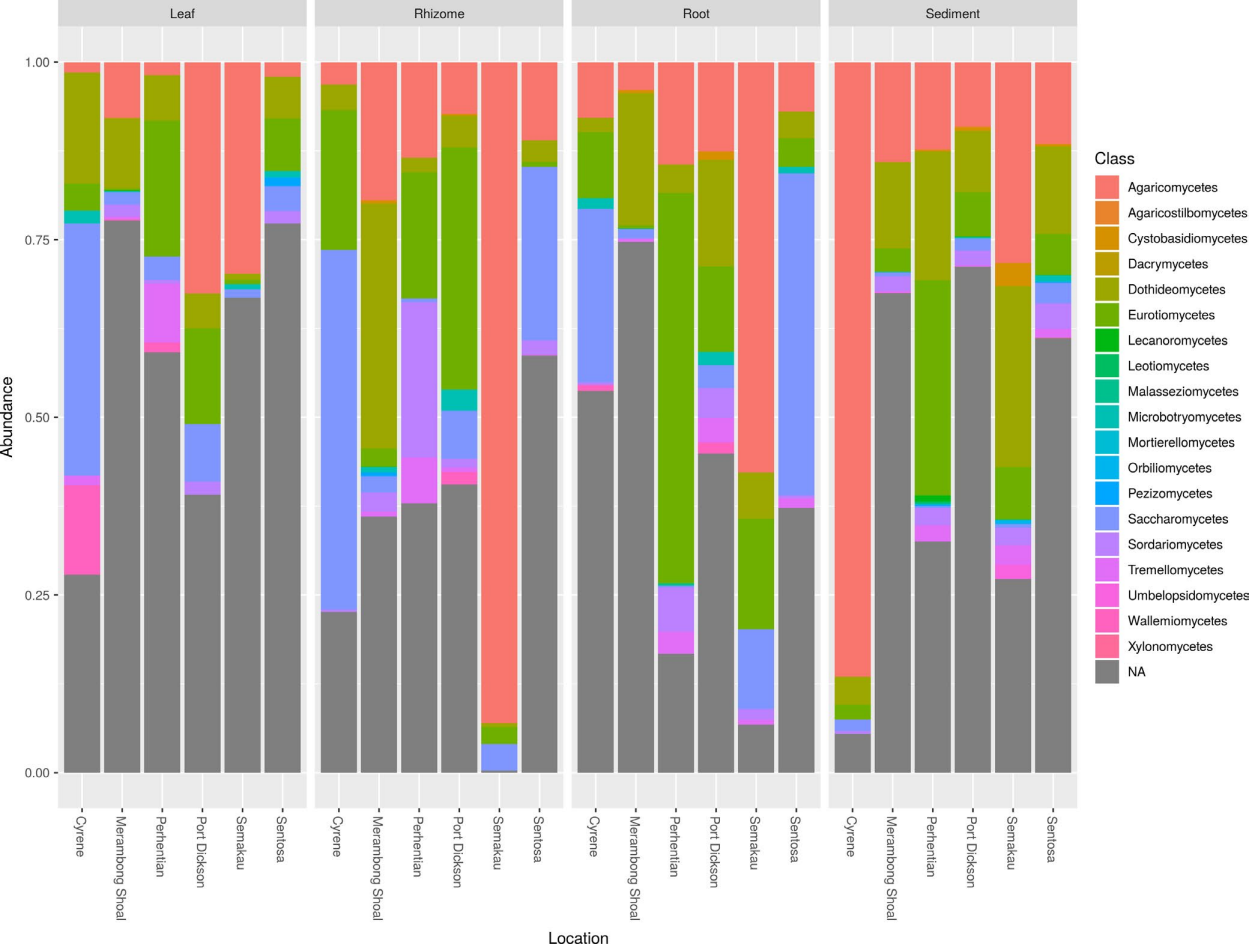
Bar plot of relative abundance in each plant part from each sample site

**Figure 7:**
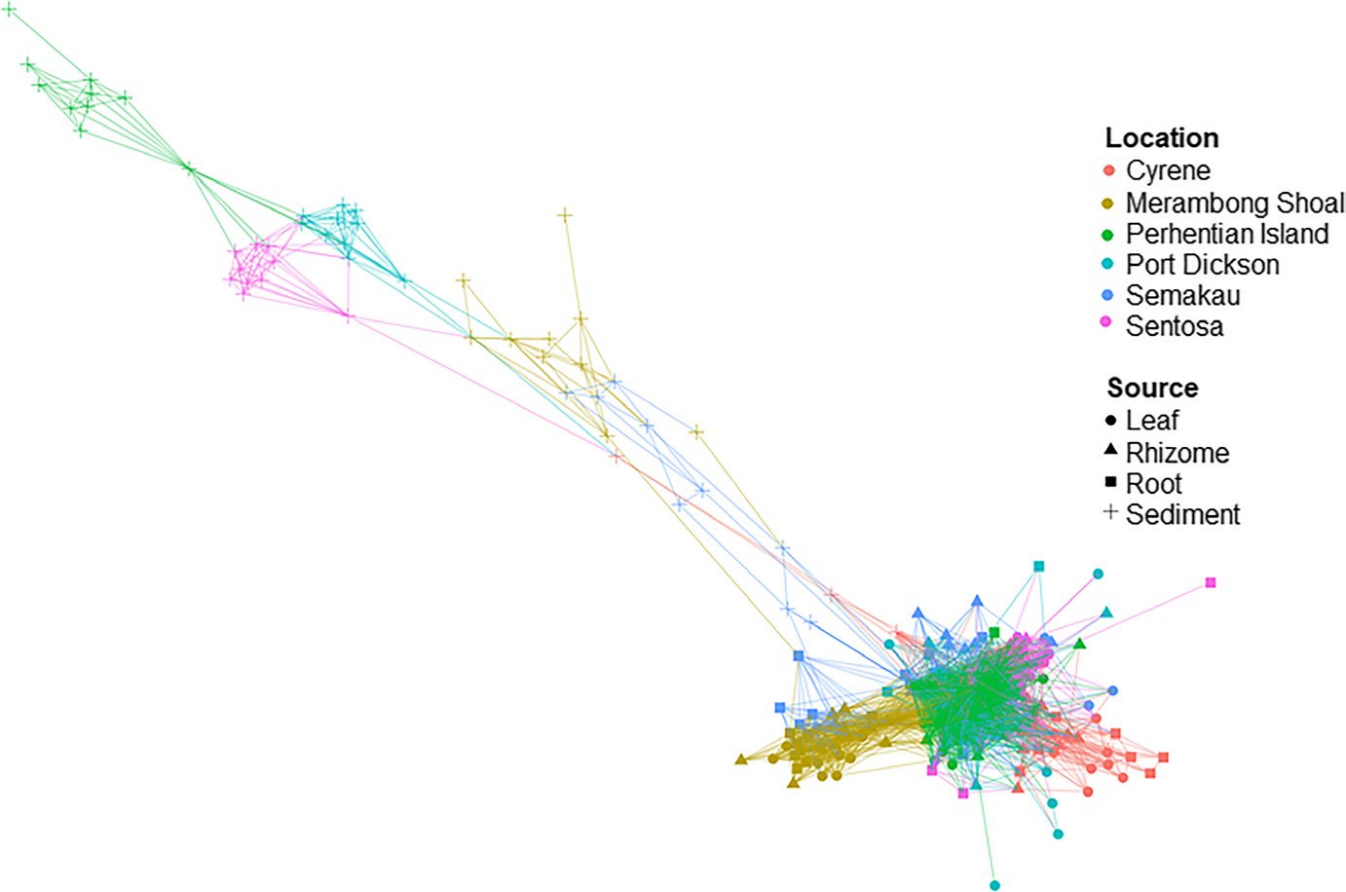
Presence–absence network plot showing connectivity of samples via shared Exact Sequence Variants (ESVs). Maximum community distance (Jaccard) was set to 0.9. Sediment samples cluster by location and share more ESVs with each other than with other sample types

Unsurprisingly, we are unable to assign a high proportion of the sequences returned here to class or even phylum level, exemplifying the limitations of our most comprehensive databases to date. This is not entirely unexpected for marine fungi given that most fungal research has focused on terrestrial environments. Similar difficulties assigning fungal taxonomy have been encountered in other understudied regions (Archer et al., 2019). All samples are dominated by fungi from phylum Ascomycota or Basidiomycota (Figure S4) and are primarily composed of fungi from the classes Dothideomycetes, Eurotiomycetes, Agaricomycetes, and Saccharomycetes (Figures 5 and 6). Despite their presence in all structures, Agaricomycetes and Dothideomycetes are found more frequently and in higher abundance in the leaves. The Wallemiomycetes, Microbotryomycetes, and Tremellomycetes all tend to be found more frequently, and at higher abundances in the belowground structures (Figure 5). Sediment samples contain the largest number of unique (not shared) ESVs, and leaves contain the fewest number of unique ESVs. Sixty‐eight ESVs are shared among all structures, and the sediment and roots share the highest number of ESVs (98) while the leaves and rhizome share the fewest ESVs of any two structures (17; Figure S5). A presence–absence network plot shows that sediment samples cluster by location and are more similar to each other than with other sample types (Figure 7), and this tendency to cluster by location is confirmed with nonmetric multidimensional scaling (NMDS; Figure S6). Plots of Shannon diversity show that Port Dickson has the most diverse fungal community, and sediment samples contain the highest fungal diversity, with rhizomes, roots, and leaves all showing a similar level of diversity (Figures S7 and S8).

## 4 DISCUSSION

Barriers to dispersal in tropical marine ecosystems are generally assumed to be few, and those that have been described are thought to be spatially and temporally permeable (Rocha, Craig, & Bowen, 2007). For example, transport into relatively isolated areas has been facilitated on occasion by infrequent large‐scale weather events such as the El Niño (Baums, Boulay, Polato, & Helberg, 2012). The permeable nature of these barriers and the high dispersal potential of most marine organisms should result in homogeneous populations (Rocha & Bowen, 2008). Similarly, with few barriers to dispersal, fungal communities could be expected to show little differentiation over large distances. However, just as work on assumed highly dispersive marine macroorganisms has revealed biogeographic patterns contrary to the expectation of homogeneity (Rocha & Bowen, 2008), there is increasing evidence that this is also the case for microorganisms (Wainwright, Afiq‐Rosli, Zahn, & Huang, 2019; Wainwright, Bauman, Zahn, Todd, & Huang, 2019). For example, fungal communities associated with the widespread seagrass *Syringodium isoetifolium* from across the Indonesian archipelago are highly differentiated on either side of Wallace's line (Wainwright, Zahn, Arlyza, & Amend, 2018). Likewise, Vincenot et al. (2017) report strong geographic structuring in a basidiomycete that disperses via aerial spores. These findings refute the expectation that species with high dispersal potential will show limited differentiation throughout their ranges. More generally, the presence of genetic and/or community structure suggests either (a) barriers to dispersal exist, (b) dispersal is limited, (c) habitat differences are present, or (d) any combination of these factors are present to drive differentiation. Reinforcing these ideas, Tedersoo et al. (2014) show strong fungal biogeographic patterns that are thought to be a consequence of dispersal limitation and habitat differences. Specifically here, we show evidence that fungal communities associated with the seagrass *E. acoroides* can be significantly differentiated by sampling location, and moreover uncover a weak but significant pattern of distance decay, meaning that samples closer in proximity have more similar communities than those farther away.

Finding geographic patterns in species that have the potential to disperse great distances should not come as a great surprise. Many marine species have a highly dispersive larval phase which can remain in the water column for multiple weeks to over 1 year (Cowen, Gawarkiewicz, Pineda, Thorrold, & Werner, 2007). This pelagic larval duration (PLD) was originally, and logically, assumed to correspond negatively with geographic structure due to the potential for high gene flow (Grantham, Eckert, & Shanks, 2003; Scheltema, 1971). But this is not generally the case, and PLD is now recognized as a poor predictor of dispersal potential and, consequently, gene flow (Shanks, 2009; Shanks, Grantham, & Carr, 2003; Weersing & Toonen, 2009). The same assumptions were proposed for fungi, that if conditions are right, fungal species will eventually arrive and colonize via dispersal (de Candolle, 1820), or as Baas‐Becking quipped in 1934, “everything is everywhere and the environment selects” (O'Malley, 2008). However, work applying molecular techniques has shown this is not the case for terrestrial fungal distributions, which reflect known barriers to dispersal (i.e., large expanses of ocean or mountains), suggesting dispersal limitations could be a factor shaping fungal biogeographic patterns (Peay, Bidartondo, & Arnold, 2010), and everything is actually not everywhere.

It is reasonable to suggest that habitat differences coupled with dispersal limitations are driving the differences, we observe in the fungal communities associated with *E. acoroides* in Singapore and Malaysia. Peay et al. (2010) discuss work showing that 95% of all spores in terrestrial fungal species fell within 45 cm of the source that produced them. If this is the case, then dispersal limitations could have profound effects on fungal biogeography. Given the viscous nature of water in comparison to air, a similar scenario is not expected to account for the significant differences and distance–decay relationship we observe in marine fungal assemblages in general. For seagrass‐associated fungi, however, dispersal limitation may have a role in structuring fungal communities. Seagrass beds are generally exposed to air during low tides and then submerged in water during the incoming tide. The outgoing tide presents an opportunity for dispersal, but countering this is the incoming tide that potentially returns fungal spores close to the location they originated, therefore restricting dispersal. A similar phenomenon has been recognized in marine larvae for over 50 years, whereby larvae are transported away from the intertidal on outgoing tides but return with the flooding tide (Carriker, 1951). This process is thought to promote local retention in species that have the potential to disperse great distances (Carriker, 1951; Cronin & Forward, 1979). Further limiting this potential for dispersal is the possibility that spores become trapped on, and under the blades of wet seagrass as the tide recedes.

The idea that habitat differences in the marine environment can drive population differentiation has been invoked to explain genetic structuring and the high diversity of coral reefs in Southeast Asia (Benzie, 1999; Bowen, Rocha, Toonen, Karl, & Laboratory, 2013; Palumbi, 1994; Sato et al., 2017; Wainwright, Arlyza, & Karl, 2019). Correspondingly, we suggest that differences in habitat type work in conjunction with dispersal limitations to structure fungal communities. An environmental cline exists as one moves in a south to north direction from Singapore through Malaysia. Amiruddin, Ibrahim, and Ismail (2011) show a gradual increase in salinity and dissolved oxygen with increasing latitude throughout the Straits of Malacca. Clinal effects over similar distances do structure fungal communities (Goldman et al., 2016), and we suggest that the environmental gradient created by increasing latitude is contributing to the observed community differences. This idea is supported by the significant Mantel test and multiple regression analyses on distance matrices showing that locations close to each other have similar fungal communities. Working in tandem with this environmental cline are probable habitat and environmental differences between eastern and western Peninsular Malaysia. Peninsular Malaysia is split in two by the Titiwangsa Mountain range, a suture zone that runs in a north to south direction for the near entirety of the country. The strata on either side of this range are different in composition; coastal eastern Malaysia is predominantly Carboniferous strata, while coastal western Malaysia is mostly Permian (Hutchinson, 2014). Given this mountain range has a maximum elevation >2,000 m and the differences in coastal strata, it is not unreasonable to suggest that environmental and physical differences occur on either side of this range. Differences in substrate chemistry (i.e., pH, organic and inorganic carbon content etc.) can significantly alter fungal community composition (Goldman et al., 2016) and it is possible that similar factors are contributing to the differences we see. We speculate that limited dispersal together with the differences in habitat are responsible for creating the patterns we observe. Additionally, microbial communities are influenced by distance from human disturbance (Jessen et al., 2013; Morrow, Moss, Chadwick, & Liles, 2012), the marine environment of Singapore is in close proximity to an urban population of more than 5.6 million people (http://www.singstat.gov.sg), and several major oil refineries and petrochemical facilities are located on offshore islands. It is extremely likely the unique environment of Singapore influences the fungal communities associated with the samples collected here.

All sampling locations and plant structures are primarily dominated by fungi from the classes Dothideomycetes, Eurotiommycetes, Agaricomycetes, and Saccharomycetes. Fungi from these classes are frequently observed in marine environments and are often associated with seagrasses (Gnavi, Ercole, Panno, Vizzini, & Varese, 2014). The prevalence of these classes in marine environments is thought to be a consequence of the efficient adaptations these fungi have evolved to marine environments. For example, spores of these taxa have appendages that enhance entrapment and adherence to marine substrate surfaces (Prasannarai & Sridhar, 2001; Vijaykrishna, Jeewon, & Hyde, 2006), further limiting dispersal.

The highest number of unique ESVs is found in the sediment. This was expected given that the majority of the currently described fungal species at some point in their life cycle occur in soil environments (Bridge & Spooner, 2001). Correspondingly, belowground structures contain more unique ESVs than leaves. The higher diversity of ESVs found in belowground structures is probably a consequence of the way that microbes are recruited by hosts (i.e., recruitment comes from the surrounding environment). Because soil is an acknowledged reservoir of fungal diversity, we see a correspondingly high diversity of fungi in belowground structures in comparison to those above ground (e.g., leaves).

The importance of fungi in promoting and maintaining plant health is widely acknowledged in terrestrial ecosystems (Ankati & Podie, 2018; Delavaux et al., 2019; Mayer, 1989). Seagrasses are marine plants and we believe it would be prudent to consider the symbiotic fungi in any seagrass restoration programme. For example, we demonstrate a significant distance–decay relationship, meaning that fungal communities are more similar to one another over short distances (i.e., close neighbors are more likely to share more of the same fungal taxa than distant sampling sites). Because of this, and like Hammerli and Reusch (2002), Fonseca (2011), and Sinclair et al. (2013) we advocate that, whenever possible, transplants should come from sources that are close to the recipient site. This would maximize similarities in fungal communities, and potentially increase conservation and restoration success (Zahn & Amend, 2017).

The methods used here are now relatively routine, becoming more cost‐effective and much more accessible, so the integration of work examining the distributions of fungal communities associated with seagrasses to enhance conservation programmes is a real possibility. Additionally, seagrass transplants and seeds could be screened for microbial pathogens that drastically reduce the success rates of seagrass conservation schemes (van Bogaert et al., 2019; Govers et al., 2016). This would also help prevent the inadvertent spread of pathogens to other regions. We encourage the incorporation of microbial community dynamics into seagrass conservation projects and hope that doing so will help maximize their chances of success.

## CONFLICT OF INTEREST

The authors declare no conflicts of interests.

## AUTHORS CONTRIBUTION

All authors contributed equally.

## ETHICAL APPROVAL

All applicable permits, international, national, and/or institutional guidelines required to perform the work were followed. Collections from Malaysia were made under permit JTLM 630‐7Jld. 9(9) and from Singapore under permit numbers NP/RP 18‐035 & NP/RP 18‐035a.

## Data Availability

All analysis code and outputs for this project, including our taxonomic reference database can be found at https://github.com/gzahn/Enhalus_Fungi, and all raw sequences associated with this work have been deposited at the National Centre for Biotechnology Information under the BioProject Accession PRJNA517736.
